# Prediction of obstructive coronary artery disease in people living with HIV: value of machine learning incorporating HIV-specific factors

**DOI:** 10.3389/fmed.2025.1661990

**Published:** 2025-09-16

**Authors:** Hui Liu, Haibo Ding, Yue Zheng, Yue Li, Yang Yu, Zhaodi Geng, Jie Zhou, Huaibi Huo, Han Li, Xin Peng, Zhaoxin Tian, Xiaolin Li, Hong Shang, Ting Liu

**Affiliations:** ^1^Department of Radiology, The First Hospital of China Medical University, Shenyang, China; ^2^Department of CT, Shaanxi Provincial People’s Hospital, Xi’an, China; ^3^State Key Laboratory for Diagnosis and Treatment of Infectious Diseases, NHC Key Laboratory of AIDS Prevention and Treatment, National Clinical Research Center for Laboratory Medicine, The First Hospital of China Medical University, China Medical University, Shenyang, China; ^4^Key Laboratory of AIDS Immunology, Chinese Academy of Medical Sciences, Shenyang, China; ^5^Key Laboratory of AIDS Immunology of Liaoning Province, Shenyang, China; ^6^Siemens Healthineers China, Shanghai, China; ^7^Department of Radiology, Jing'an District Centre Hospital of Shanghai, Fudan University, Shanghai, China; ^8^Department of Radiology, The Third People's Hospital of Chengdu, Chengdu, China

**Keywords:** HIV, coronary artery disease, pre-test probability, coronary CTA, machine learning

## Abstract

**Objectives:**

To explore the value of machine learning (ML) model in conjunction with HIV-specific risk factors to predict obstructive coronary artery disease (CAD) (≥50% stenosis) on coronary CT angiography (CTA) in the asymptomatic people living with HIV (PLWH).

**Methods:**

In this cross-sectional study, we prospectively analyzed 304 PLWH without chest pain (age 48 ± 11 years, 91% males). The dataset was randomly divided into training and held-out test sets in an 8:2 ratio. The ML model established by random forest was compared with traditional models, including CAD consortium clinical score, CONFIRM score, and Genders clinical model, as well as logistic regression model. The coronary artery calcium score (CACS) was added to the above five models to establish new models. Predictive performance of the models was evaluated according to Delong test.

**Results:**

Obstructive CAD occurred in 64 of 304 PLWH (21%). The ML model (AUC of 0.946) had the highest discrimination for obstructive CAD compared with above models (AUC of 0.734, 0.736, 0.737, and 0.782, respectively; *p* < 0.05 for all comparisons). ML model showed the best calibration and clinical decision-making capability. Moreover, the ML model showed the best predictive performance compared with models after adding the CACS (AUC of 0.772, 0.740, 0.742, 0.750, and 0.798, respectively; *p* < 0.05 for all comparisons).

**Conclusion:**

The ML model incorporating cardiovascular risk factors and HIV-specific factors can more accurately estimate the pretest likelihood of obstructive CAD in PLWH than traditional models. ML improves risk stratification in HIV populations and may help guide management.

## Introduction

The AIDS epidemic has been a global public health issue for more than 40 years and has resulted in approximately 84 million infections and 40 million deaths (1). Antiretroviral therapy (ART) can slow the progression of the disease and prolong the life of an infected person by suppression of viral replication and immune restoration (2, 3). Persistent immune activation despite ART, HIV-specific risk factors, and the aging PLWH population have been associated with a higher risk of cardiovascular disease (CAD), compared with the general population (4–6).

Coronary CT angiography (CTA) is widely accepted and has become the main imaging test for the diagnosis of CAD (7). However, in daily clinical practice, a significant number of individuals undergoing coronary CTA have minimal or no CAD, which results in additional radiation exposure and suboptimal cost-effectiveness (8). The European guideline has therefore recommended the performance of pre-test probability assessments to guide clinical decisions on whether diagnostic testing should be deferred or conducted and whether the initial test should be non-invasive or invasive (9).

Existing conventional prediction models of obstructive CAD, including the CAD consortium clinical score, CONFIRM registry score (CRS), and genders clinical model (GCM) are only applicable to non-HIV populations with chest pain (9–11). However, these general population-derived models are not directly applicable to the asymptomatic high-risk PLWH population and HIV-specific risk factors contributing to CAD.

Artificial intelligence and machine learning (ML) have shown promise to improve risk assessment in various clinical scenarios, with iterative algorithms having an advantage over linear algorithms for the internal relations among confounding factors. Applications of ML have not only been limited to medical image reading, but have also been widely used in medical data analysis (12). Furthermore, the integrated use of clinical and imaging data to predict clinical outcomes shows great promise (13).

Accordingly, we sought to explore the value of a machine learning model combining cardiovascular risk factors, hematologic indicators, and HIV-specific factors to predict the risk of obstructive CAD in PLWH more accurately.

## Materials and methods

### Study population

**Figure 1:**
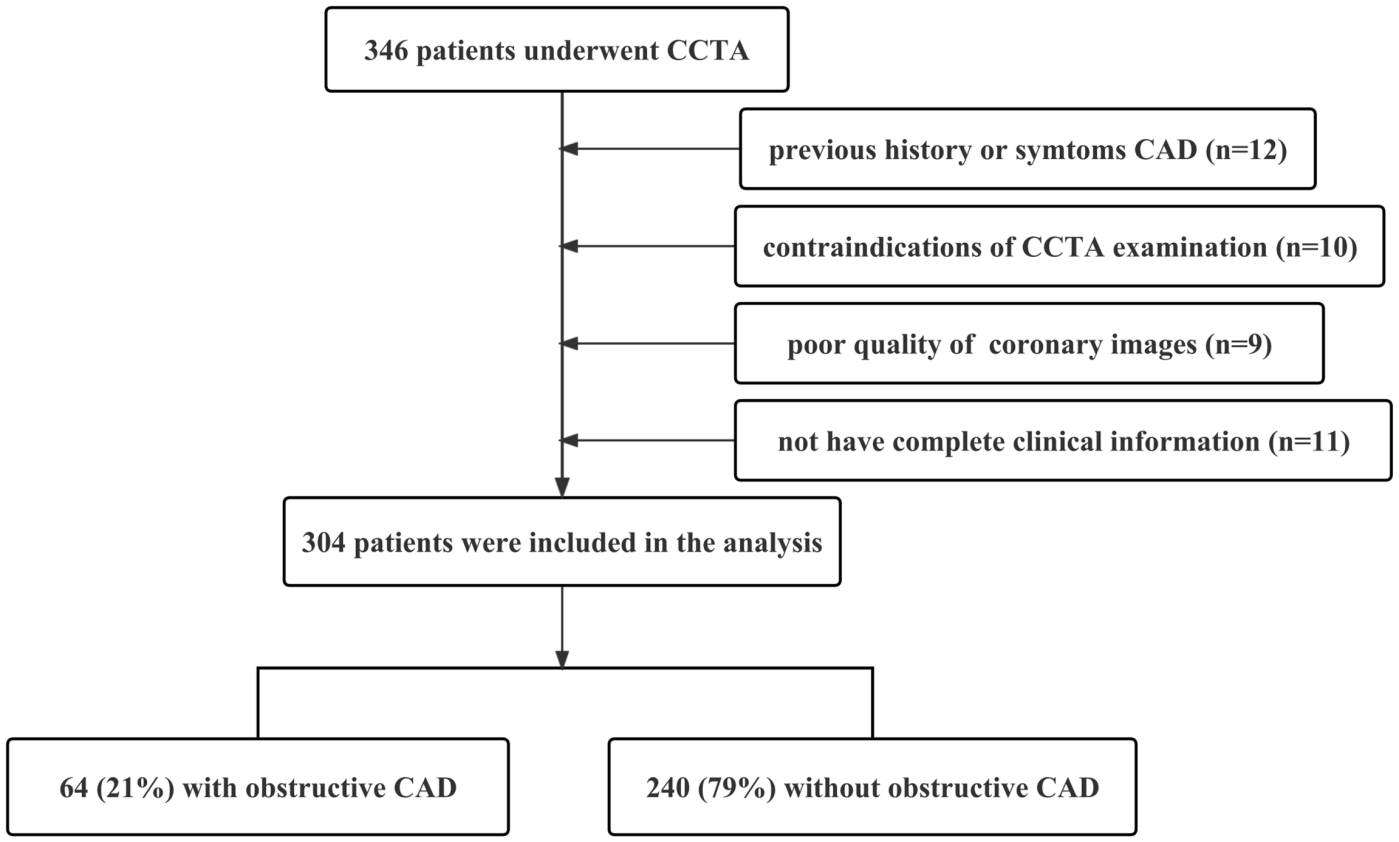
Flowchart of patient recruitment with inclusion and exclusion criteria.

In this cross-sectional study, we enrolled 346 PLWH who underwent 64-detector row coronary CTA evaluation between December 2019 and June 2023. The inclusion criteria for PLWH included (1) diagnosis of HIV infection, (2) age between 20 and 80 years, (3) undergoing ART. The exclusion criteria included: (1) previous history or symptoms CAD, (2) contraindications of CCTA examination, (3) poor quality of coronary images, and (4) not have complete clinical information (Figure 1). The final dataset contained no missing values. Eventually, 304 patients were enrolled for final analysis. Fifty of these patients have been used for the earlier MRI study, but the current CT study is analyzing more patients with a completely different goal and result (14). This study was approved by the local Ethics Review Board and conducted in accordance with the Declaration of Helsinki.

### Data collection

Prior to the coronary CTA examination, demography (age, sex), clinical information (cardiovascular risk factors, antihypertensive therapy, HIV acquisition mode, hepatitis C, intravenous drug use, the time since HIV diagnosis), hematologic and immunological factors (estimated glomerular filtration rate, HIV viral load, current CD4^+^ T-cell count, CD8^+^ T-cell count, and nadir CD4^+^ T-cell count, CD4^+^/CD8^+^), and treatment-related factors (duration on ART, ART regimen) was prospectively collected for all PLWH participants.

### Coronary CTA scanning protocol and image analysis

Coronary CTA was performed using a third-generation dual-source CT (SOMATOM Force, Siemens Healthcare, Forchheim, Germany) and 45 to 100 mL of intravenous contrast (Iopamidol Injection, 370 mg/mL, Bracco Si, Shanghai, China) at a flow rate of 4.0–5.0 mL/s. Retrospective electrocardiographic gating was used in all of the participants. The main scan parameters were as follows: tube voltage = 100 kV, tube current = 300–400 mas, slice thickness = 0.75 mm, detector collimation = 2 mm × 64 mm × 0.6 mm, and matrix = 512 × 512. A non-contrast CT examination was performed for coronary calcium scoring.

All the scans were sequentially analyzed and reviewed on a workstation (Syngo.via version VB20, Siemens Healthineer, Forchheim, Germany) by two radiologists (with three and fifteen years of experience, respectively) using axial images and multiplanar reconstructions. The coronary segment was analyzed by using the 16-segment American Heart Association definitions from 1975 (15). The outcome was the presence of obstructive CAD demonstrated by coronary CTA, which has been shown in previous studies to have a high diagnostic accuracy for obstructive CAD (16, 17). Obstructive CAD was defined as at least 1 coronary segment with a lesion of ≥50% luminal stenosis in diameter (18). Coronary artery calcium score (CACS) measurement was performed using the Agatston method (19). Observations between the two readers were analyzed for interobserver agreement.

### Model construction

We developed a total of 11 models to predict obstructive CAD. There are three traditional conventional pre-test probability models of coronary CTA. All our patients were asymptomatic, so clinical symptoms could not be included as a feature in the traditional model. In addition to gender, age, hypertension, diabetes, and smoking status, CAD consortium clinical score requires hyperlipidemia and BMI, CRS requires cardiovascular family history, and GCM requires hyperlipidemia. The logistic regression (LR) model was built using all available features, including traditional cardiovascular and HIV-specific variables. Moreover, we added CACS to above four models to create four additional models: CAD + CACS, CRS + CACS, GCM + CACS, and LR + CACS. A CACS model using only the calcium score was included for comparison.

Furthermore, three ML models were developed based on the random forest algorithm. Model 1 incorporated all available clinical, laboratory, and HIV-specific variables. After adding CACS, Model 2 was reconstructed with renewed feature selection and hyperparameter tuning. To evaluate the incremental value of CACS, Model 3 retained the features and hyperparameters selected in Model 1, with CACS added as an additional variable.

Consistent with the 2019 ESC Guidelines, our machine learning model incorporates multiple cardiovascular risk factors to estimate the likelihood of obstructive CAD (20). To facilitate clinical risk interpretation, we adopted the risk stratification thresholds proposed in the 2013 ESC Guidelines based on pretest probability: low risk (<15%), intermediate risk (15–85%), and high risk (>85%) (21). Patients with a low pretest probability are less likely to benefit from additional testing, while those with an intermediate probability are most likely to benefit from initial noninvasive testing.

### Machine learning

Random forest algorithm was performed to model our data. The original dataset was randomly divided into training and held-out test sets in an 8:2 ratio to maintain the ratio of obstructive to non-obstructive CAD in both subsets. In addition, model hyperparameters are optimized by five-fold cross-validation, which repeats five iterations by randomly splitting the training set into five equal-sized subsets, four were used for inner training and one was used for inner test. The employ of cross-validation in the training set can be beneficial as its primary use is to empirically determine the optimal model hyperparameters without resorting to the test set (22). Subsequently, the optimal hyperparameters are applied to evaluate the model in the test set (maximum tree depth: 4; number of trees: 5; random seed: 2175). Finally, the area under the curve (AUC) and the associated 95% confidence interval were used to measure the classification performance of the ML model. Feature selection is performed using the Boruta algorithm. The feature selection and modeling process were performed in the training set, and the independent test set was only used to evaluate the performance of the model and was not involved in the training process.

### Statistical analysis

Continuous variables were expressed as median with 25th–75th percentile interquartile range (IQR), depending on the normality of distribution assessed by the Kolmogorov–Smirnov test. Categorical variables were presented as frequencies and percentages. Comparisons between groups were carried out using an independent t-test or the Mann–Whitney U test for normally and non-normally distributed continuous variables and the chi-square test or Fisher’s exact test for categorical variables, as appropriate.

The discriminative power of Model 1 was compared with the 4 traditional models and LR model, respectively, using receiver-operating characteristic analysis and pairwise comparisons according to Delong et al. (23). *p* < 0.05 indicated statistical significance between the performance of the models. Calibration curves were drawn to explore the agreement between the observed outcome frequencies and predicted probabilities of the models in the testing group. Decision curve analysis was used to assess the clinical utility of these models by quantifying the net benefits at different risk threshold probabilities in testing set.

All statistical analyses were carried out using IBM SPSS software (V26.0; IBM Corporation, Armonk, New York, USA) and R software (version 4.3.1).

## Results

**Figure 2:**
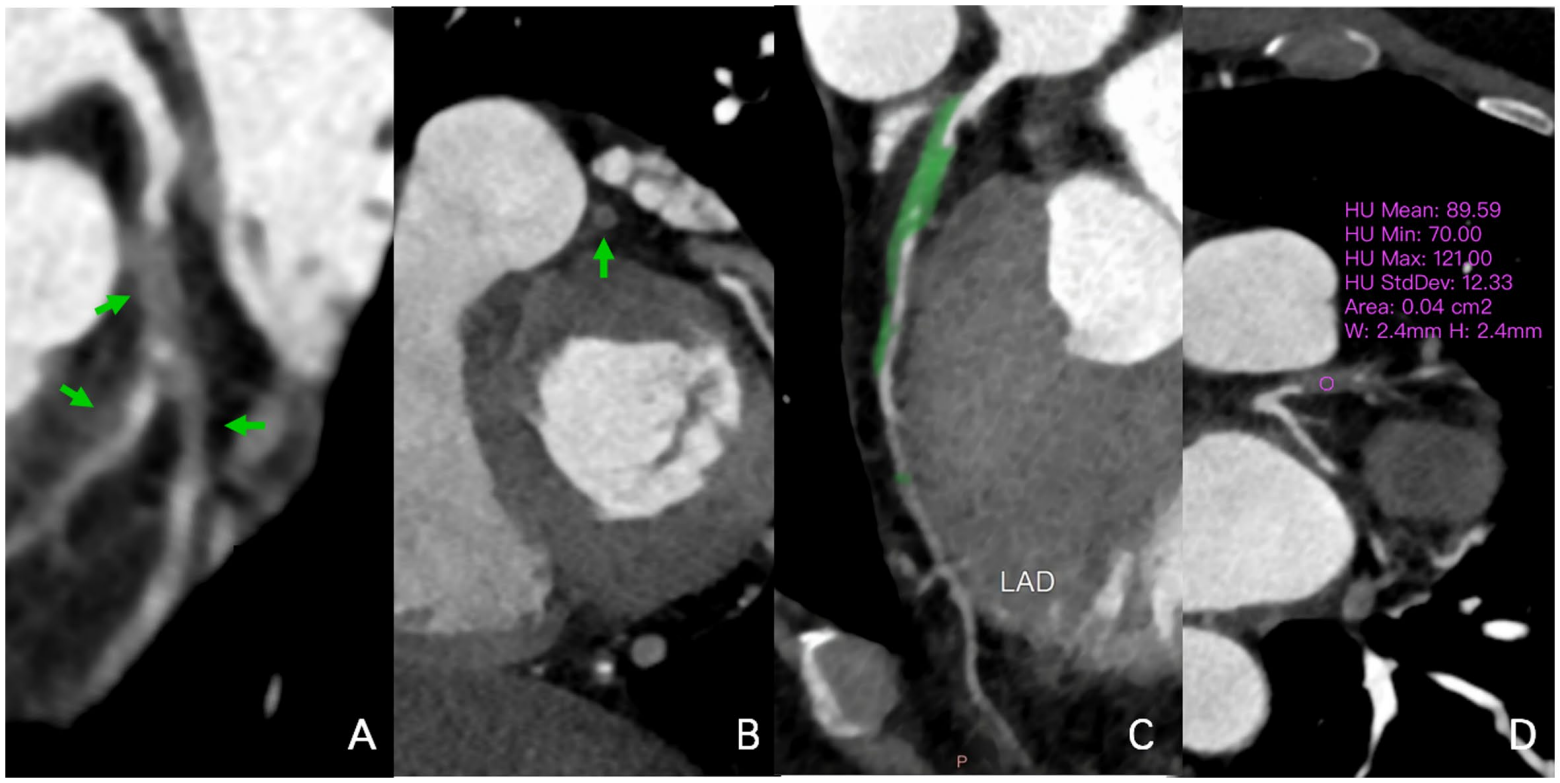
A 58-year-old man infected with HIV. In the multiplanar reconstruction of coronary CTA **(A–D)**, non-calcified plaques (70-121HU) were seen in the proximal segment of the LAD and the beginning of the first diagonal branch, with severe lumen stenosis (90–99%). Noncalcified plaque also was seen in the middle segment of the LAD with mild lumen stenosis (40–50%). HIV, human immunodeficiency virus; CTA, CT angiography; HU, Hounsfield units; LAD, left anterior descending artery.

A total of 304 patients met the inclusion criteria and were included in the analysis. The mean age of the patients was 48 ± 10 years, and 91% were male. The occurrence of obstructive CAD was 21% (64/304) within the studied cohort (Figure 2). The clinical characteristics are listed in Table 1. The presence of obstructive CAD was significantly associated with age, hypertension, use of antihypertensive drugs, total cholesterol, low-density lipoprotein, hyperlipidemia, and estimated glomerular filtration rate. Among HIV-related indicators, nadir CD4^+^ T-cell count and duration of antiretroviral drug therapy were significantly associated with obstructive CAD.

**Table 1:** Patient characteristics.

Characteristic	All participants (*n* = 304)	With obstructive CAD (*n* = 64)	Without obstructive CAD (*n* = 240)	*p*-value
Male sex, *n* (%)	277 (91.1)	60 (93.8)	217 (90.4)	0.405
Age (years), median (IQR)	49.0 (41.0–56.0)	55.5 (49.0–61.0)	47.0 (40.0–53.0)	<0.001
Body mass index (kg/m^2^), median (IQR)	23.0 (21.5–25.3)	23.2 (21.7–25.5)	22.9 (21.4–25.2)	0.552
Systolic pressure (mmHg), median (IQR)	125.0 (115.0–135.0)	135.0 (121.0–144.8)	126.0 (120.0–138.0)	0.001
Diastolic pressure (mmHg), median (IQR)	80.0 (78.0–90.0)	88.0 (80.0–96.0)	81.5 (78.0–90.0)	0.001
Hypertension, *n* (%)	39 (12.8)	14 (21.9)	25 (10.4)	0.015
Antihypertensive drugs, *n* (%)	26 (8.6)	10 (15.6)	16 (6.7)	0.023
Diabetes mellitus, *n* (%)	20 (6.6)	7 (10.9)	13 (5.4)	0.113
Total cholesterol (mmol/L), median (IQR)	4.7 (4.1–5.3)	4.9 (4.5–5.7)	4.6 (4.0–5.2)	0.004
HDL cholesterol (mmol/L), median (IQR)	1.1 (1.0–1.3)	1.1 (0.9–1.3)	1.2 (1.0–1.4)	0.971
LDL cholesterol (mmol/L), median (IQR)	2.8 (2.3–3.5)	2.9 (2.2–3.8)	2.8 (2.3–3.4)	0.103
Triglyceride (mmol/L), median (IQR)	1.8 (1.1–2.7)	1.9 (1.3–3.4)	1.8 (1.1–2.6)	0.112
Hyperlipidemia, *n* (%)	193 (63.5)	48 (75.0)	145 (60.4)	0.031
Current smoking, *n* (%)	97 (31.9)	23 (35.9)	74 (30.8)	0.436
Current alcohol, *n* (%)	114 (37.5)	25 (39.1)	89 (37.1)	0.771
Family history of CAD, *n* (%)	70 (23.0)	18 (28.1)	52 (21.7)	0.276
eGFR, median (IQR)	106.7 (98.2–115.1)	99.6 (88.3–107.6)	108.0 (99.8–114.3)	<0.001
Hepatitis C seropositivity, *n* (%)	4 (1.3)	1 (1.6)	3 (1.3)	1.000
CACS, median (IQR)	0 (0–3.4)	12.4 (0–82.5)	0	<0.001
CD4^+^ count (cells/mm^3^), median (IQR)	591.5 (455.5–768.0)	580.5 (410.8–713.0)	595.0 (458.0–770.5)	0.199
CD4^+^ nadir (cells/mL^3^), median (IQR)	283.2 (177.0–377.4)	220.2 (92.5–363.3)	295.5 (201.0–378.9)	0.032
CD4^+^/CD8^+^, median (IQR)	0.8 (0.6–1.2)	0.7 (0.5–1.0)	0.8 (0.6–1.2)	0.618
Active illicit drug use, n (%)	5 (1.6)	3 (4.7)	2 (0.8)	0.065
On ART, *n* (%)				0.242
NNRTI	194 (63.8)	39 (60.9)	155 (64.6)	
IN	87 (28.6)	17 (26.6)	70 (29.2)	
PI	23 (7.6)	8 (12.5)	15 (6.3)	
Years of HIV-diagnosed, median (IQR)	7.0 (4.0–11.0)	8.0 (4.3–10.8)	7.0 (4.0–11.0)	0.577
Years of HIV-treated, median (IQR)	8.0 (5.0–11.0)	8.0 (5.0–11.0)	7.0 (5.0–9.0)	0.208
Viral load ≥50 copies/mL, *n* (%)	34 (11.2)	11 (17.2)	23 (9.6)	0.086

IQR, interquartile range; HDL cholesterol, high density lipoprotein cholesterol; LDL cholesterol, low density lipoprotein cholesterol; CAD, cardiovascular disease; eGFR: estimated glomerular filtration rate; CACS, coronary artery calcium score; ART, antiretroviral therapy; NNRTI, non-nucleoside reverse-transcriptase inhibitor; IN, integrase inhibitors; PI, protease inhibitors.

### The importance of features

**Figure 3:**
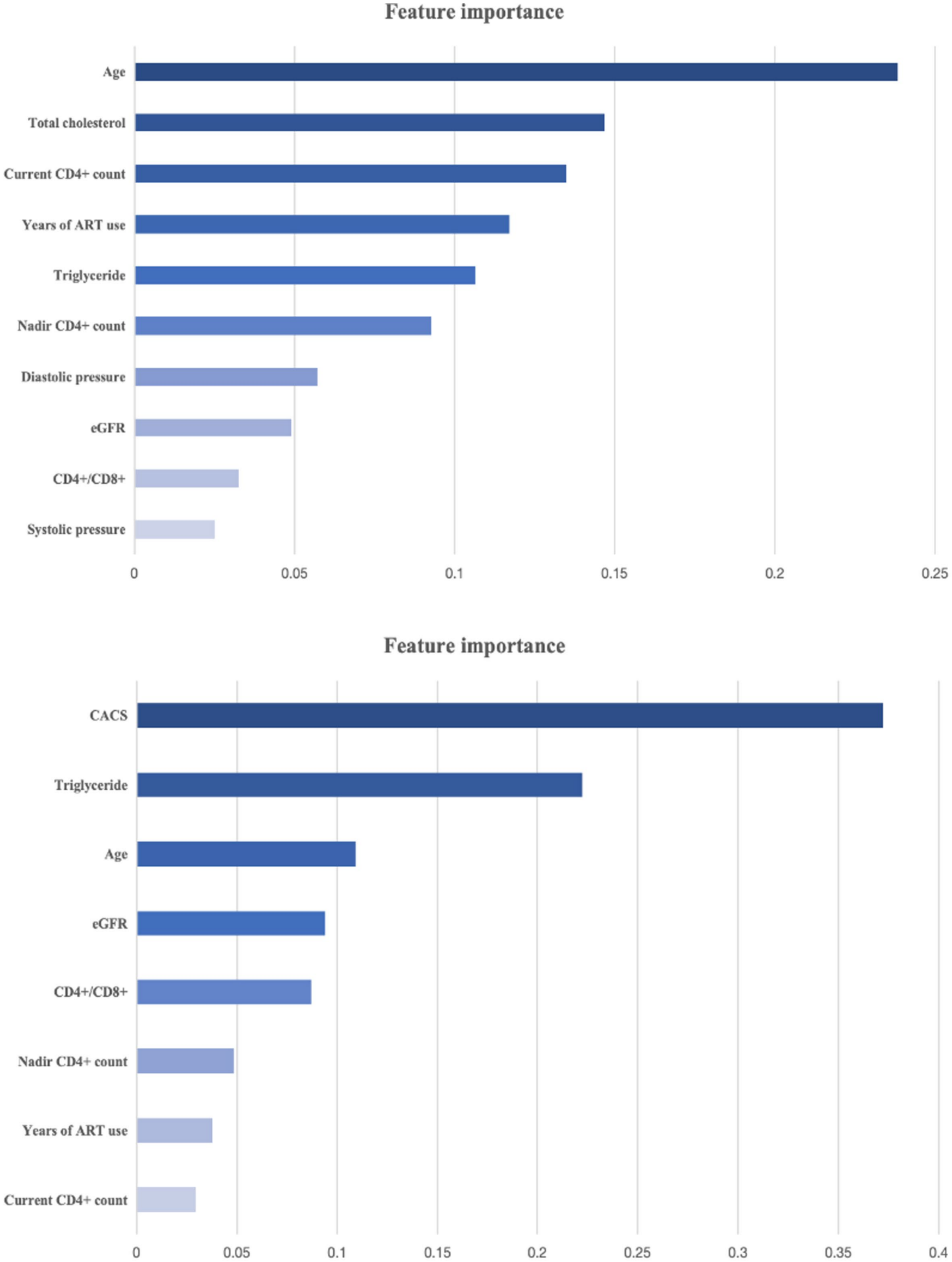
The importance ranking of features for the **(A)** Model 1 and **(B)** Model 2. The figure shows the variables added to the model after Boruta algorithm. CACS, coronary artery calcium score.

Figure 3 presents the feature importance rankings generated by the Boruta algorithm. As shown, age, total cholesterol, and current CD4^+^ T-cell count were the most predictive features in the Model 1. This was followed by years of ART use, triglycerides, nadir CD4^+^ T-cell count, and diastolic blood pressure. Interestingly, the importance of traditional cardiovascular risk factors increased with the addition of the CACS into the model, the most predictive features (after the CACS itself) were triglycerides and age followed by estimated glomerular filtration rate and CD4^+^/CD8^+^. The risk factors for obtaining models after screening are shown in Table 2.

**Table 2:** The risk factors for obtaining models.

Models	Features
CAD	age, gender, diabetes, hypertension, hyperlipidemia, smoking, BMI
CRS	age, gender, diabetes, hypertension, smoking, family history of CVD
GCM	age, gender, diabetes, hypertension, smoking, hyperlipidemia
LR Model	age, TG, nadir CD4^+^ T-cell count, diastolic pressure
CACS Model	CACS
CAD + CACS	CACS, age, gender, diabetes, hypertension, hyperlipidemia, smoking. BMI
CRS + CACS	CACS, age, gender, diabetes, hypertension, smoking, family history of CVD
GCM + CACS	CACS, age, gender, diabetes, hypertension, smoking, hyperlipidemia
LR + CACS	CACS, age, TG, nadir CD4^+^ T-cell count, diastolic pressure
Model 1	age, TC, current CD4^+^ T-cell count, years of ART use, TG, nadir CD4^+^ T-cell count, Diastolic blood, eGFR, CD4^+^/CD8^+^, systolic pressure
Model 2	CACS, TG, age, CD4^+^/CD8^+^, nadir CD4^+^ T-cell count, years of ART use, current CD4^+^ T-cell count
Model 3	CACS, age, TC, current CD4^+^ T-cell count, years of ART use, TG, nadir CD4^+^ T-cell count, Diastolic blood, eGFR, CD4^+^/CD8^+^, systolic pressure

CAD: CAD Consortium clinic score; CRS, CONFIRM score; GCM, Genders clinical model; LR, logistics regression; BMI, body mass index; CVD, cardiovascular disease; TG, triglyceride; TC, total cholesterol; ART, antiretroviral therapy; eGFR: estimated glomerular filtration rate; CACS, coronary artery calcium score.

### Discriminative power

**Figure 4:**
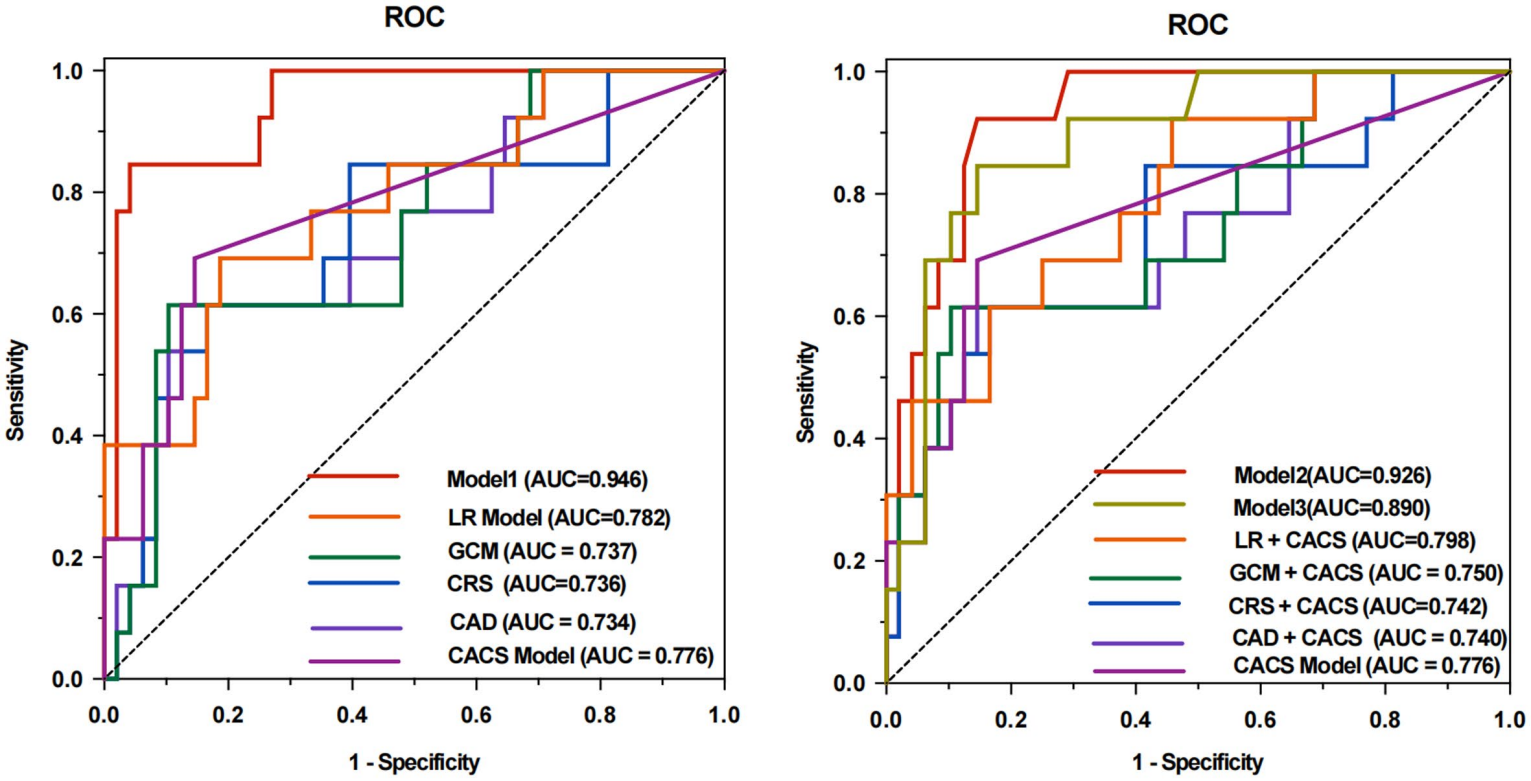
ROC analyses for predicting rapid plaque progression of all models. The area under the receiver operating characteristic curve (ROC) of Model 1 was 0.946 compared to 0.734 (*p* = 0.003) for CAD, 0.736 (*p* = 0.004) for CRS, 0.737 (*p* = 0.002) for the GCM, 0.782 (*p* = 0.008) for LR model, and 0.776 (*p* = 0.020) for CACS model **(A)**. With the addition of CACS into the Model 1, the performance of the model (Model 2, AUC of 0.926) still showed the best discriminative performance compared to 0.740 (*p* = 0.022) for CAD + CACS, 0.742 (*p* = 0.023) for CRS + CACS, 0.750 (*p* = 0.025) for the GCM + CACS, 0.798 (*p* = 0.043) for LR + CACS, and 0.776 (*p* = 0.012) for CACS model **(B)**. Without Boruta algorithm, incorporating the CACS into the feature set of Model 1 (Model 3, AUC of 0.890) did not yield a significant improvement in model performance (*p* = 0.168). CAD, CAD Consortium clinical score; CRS, CONFIRM score; GCM, Genders clinical model; LR, logistics regression; AUC, area under the ROC curve; CACS, coronary artery calcium.

The machine learning model (Model 1, AUC of 0.946), which incorporated both traditional cardiovascular risk factors and HIV-specific metrics, showed the best performance in predicting obstructive CAD compared with CAD Consortium clinical score, CRS, and GCM (AUC of 0.734, 0.736, and 0.737, respectively; *p* < 0.05 for all comparisons). In order to control for potential confounding due to algorithm differences, we additionally constructed a logistic regression (LR) model using the full feature set (LR model, AUC of 0.782; *p* < 0.05), confirming the superior predictive capability of the random forest–based Model 1 (*p* < 0.05). Furthermore, the LR model did not differed significantly from traditional models. The sensitivity, specificity, positive predictive value, negative predictive value, and accuracy for the prediction of obstructive CAD were 0.846, 0.958, 0.846, 0.958, and 0.934 for Model 1, respectively. Interestingly, Model 2, developed by adding CACS and reapplying feature selection, yielded a slightly lower AUC of 0.926 but still outperformed the CAD + CACS model, CRS + CACS model, GCM + CACS model, and LR + CACS model (AUC of 0.740, 0.742, 0.750, and 0.798; *p* < 0.05 for all comparisons). After adding CACS to the Model 1 feature set without re-optimization, Model 3 demonstrated a reduced AUC of 0.890, with no statistically significant difference compared to Model 1 or Model 2 (*p* > 0.05). Meanwhile, the performance of Model 1 and Model 2 significantly outperformed the CACS models (AUC of 0.776; *p* < 0.05), suggesting that clinical and HIV-specific features contributed more substantially to predictive performance than CACS alone. The discriminative performance of all models is shown in Tables 3, 4 and Figure 4.

**Table 3:** The Discriminative Power of all Models for Obstructive CAD.

Models	Statistical approach	AUC (95% CI)	*p*-value (compare with Model1)	*p*-value (compare with Model2)
CAD	Logistic regression	0.734 (0.605–0.839)	0.003	0.018
CRS	Logistic regression	0.736 (0.607–0.840)	0.004	0.023
GCM	Logistic regression	0.737 (0.609–0.842)	0.002	0.016
LR Model	Logistic regression	0.782 (0.658–0.878)	0.008	0.045
CACS Model	Logistic regression	0.776 (0.651–0.873)	0.020	0.012
CAD + CACS	Logistic regression	0.740 (0.612–0.844)	0.005	0.022
CRS + CACS	Logistic regression	0.742 (0.614–0.846)	0.006	0.023
GCM + CACS	Logistic regression	0.750 (0.623–0.852)	0.006	0.025
LR + CACS	Logistic regression	0.798 (0.676–0.890)	0.005	0.043
Model 1	Random forest	0.946 (0.856–0.987)	\	0.571
Model 2	Random forest	0.926 (0.830–0.977)	0.571	\
Model 3	Random forest	0.890 (0.799–0.969)	0.168	0.285

CAD, CAD Consortium clinic score; CRS, CONFIRM score; GCM, Genders clinical model; LR, logistics regression; AUC, area under the ROC curve; CI, confidence interval; CACS, coronary artery calcium score.

**Table 4:** The performance of all models in the training and testing groups.

Model	Training group					Testing group				
	ACC	SEN	SPE	PPV	NPV	ACC	SEN	SPE	PPV	NPV
Model 1	0.868	0.882	0.865	0.634	0.965	0.934	0.846	0.958	0.846	0.958
Model 2	0.926	0.804	0.958	0.837	0.948	0.869	0.923	0.854	0.632	0.976
Model 3	0.926	0.706	0.984	0.923	0.927	0.853	0.539	0.938	0.700	0.882
LR Model	0.757	0.686	0.776	0.449	0.903	0.787	0.692	0.813	0.500	0.907
GCM	0.798	0.490	0.880	0.521	0.867	0.820	0.615	0.875	0.571	0.894
CRS	0.757	0.698	0.797	0.443	0.884	0.771	0.692	0.792	0.474	0.905
CAD	0.811	0.412	0.917	0.568	0.854	0.836	0.615	0.896	0.615	0.896
CACS	0.778	0.667	0.807	0.479	0.901	0.820	0.692	0.854	0.562	0.911
LR + CACS	0.807	0.725	0.828	0.529	0.919	0.623	0.923	0.542	0.353	0.963
GCM + CACS	0.720	0.882	0.677	0.421	0.956	0.836	0.615	0.896	0.615	0.896
CRS + CACS	0.794	0.706	0.818	0.507	0.913	0.787	0.615	0.833	0.500	0.889
CAD + CACS	0.753	0.784	0.745	0.449	0.929	0.803	0.615	0.854	0.533	0.891

ACC, accuracy; SEN, sensitivity; SPE, specificity; PPV, positive predictive value; NPV, negative predictive value. CAD, CAD Consortium clinic score; CRS, CONFIRM score; GCM, Genders clinical model; LR, logistics regression; CACS, coronary artery calcium score.

### Calibration and clinical decision

**Figure 5:**
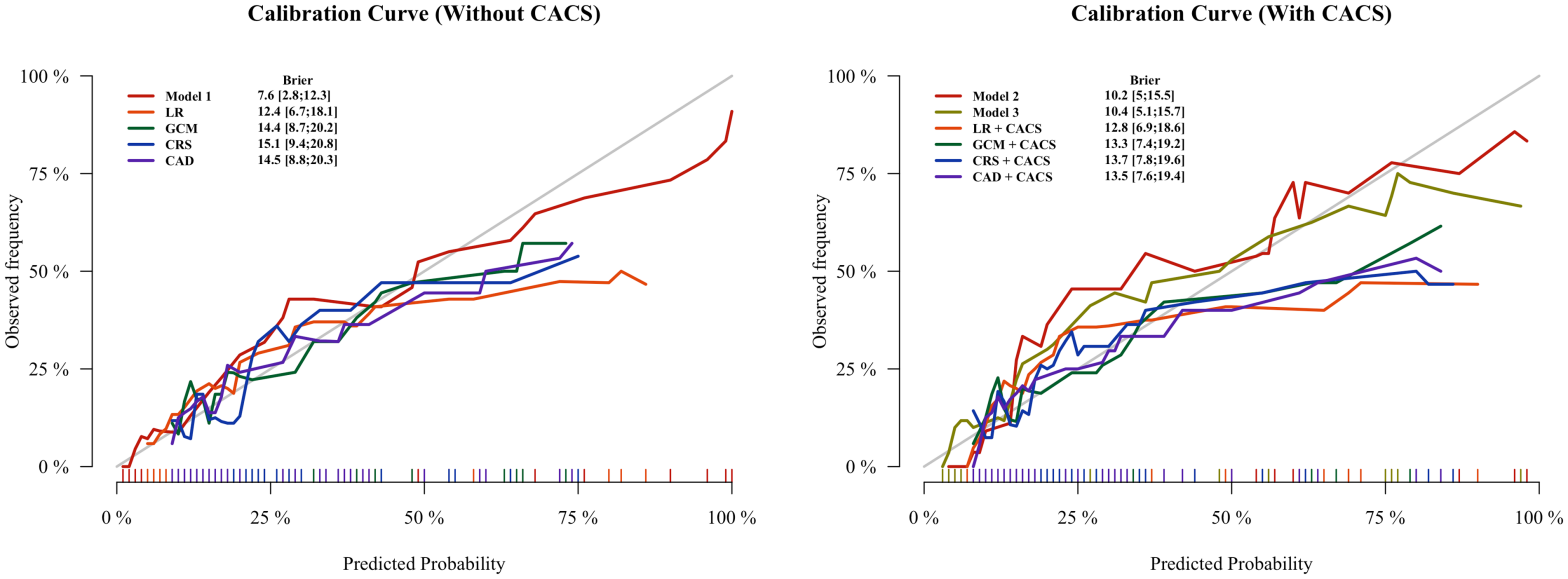
Calibration plots for all methods of estimating pretest probability of obstructive coronary artery disease in the test groups. The lowest Brier scores (on a scale ranging from 0 to 1) for the Model 1 is 0.076, followed by Model 2 with 0.102. The Brier score calculates the difference between the predicted and observed probability for occurrence of obstructive CAD, with values closer to 0 indicating better calibration. CAD, CAD Consortium clinical score; CRS, CONFIRM score; GCM, Genders clinical model; LR, logistics regression; CACS, coronary artery calcium score.

**Figure 6:**
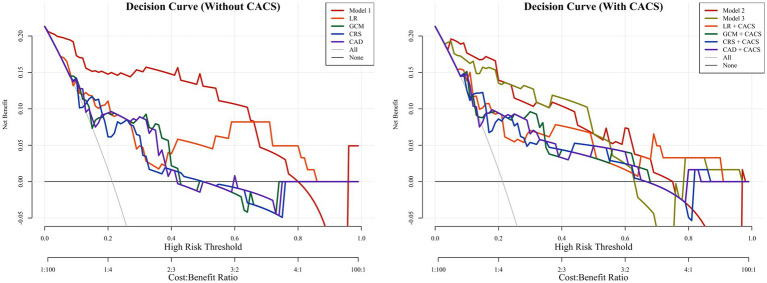
The decision curves of all methods in the test groups. The decision curves showed that if the threshold probability was between 0 and 70%, using the Model 1 to predict probability of obstructive coronary artery disease has more benefit than other models. With the addition of CACS, the Model 2 and Model 3 showed better predictive ability between threshold probabilities of 0 ~ 70%. CAD, CAD Consortium clinical score; CRS, CONFIRM score; GCM, Genders clinical model; LR, logistics regression; CACS, coronary artery calcium score.

In both groups, the calibration curves of Model 1 and Model 2 showed the best calibration of the predicted probability with the true probability (Brier scores of 0.076 and 0.102, respectively) (Figure 5). The clinical usefulness is shown as decision curve in Figure 6. The ML Models demonstrated superior net benefit in decision curve analysis when the threshold probability ranged from 0 to 70%.

## Discussion

This cross-sectional study explores the prediction of the probability of obstructive CAD in a cohort of PLWH by using machine learning in conjunction with clinical risk factors and HIV-specific risk markers. We found that HIV-associated immunologic markers are strongly associated with predicting the prevalence of obstructive CAD in populations with well-controlled HIV disease. This study extends our understanding of obstructive CAD in PLWH, demonstrating the improved performance of machine learning models constructed with the random forest algorithm.

We demonstrated that machine learning models were more applicable to HIV-infected individuals than traditional models with only traditional risk factors. Existing conventional prediction models are only used in the symptomatic general population, with the prevalence of obstructive CAD ranging from 18–24% in previous studies (24–26). However, in this study, stenosis greater than 50% in asymptomatic HIV patients accounted for 21%, indicating some of the PLWH had already suffered from obstructive CAD during the subclinical period without timely examination and further treatment, which may lead to major adverse cardiac events. Therefore, early diagnosis and treatment targeting high-risk populations hold great clinical relevance. However, the use of the LR algorithm did not improve the performance of the model even when HIV-specific factors were added. This suggests that there is a non-linear relationship among HIV-specific factors, and that logistic algorithms alone are unable to make better predictions of outcomes resulting from complex and multiple factors (27, 28). Random forests not only explore HIV-specific markers that have an impact on CAD occurrence through an iterative algorithm, but also have a higher predictive ability for obstructive CAD than traditional or LR models (AUC value of 0.946 and accuracy of 0.934).

The mechanisms underlying the increased prevalence of CAD in PLWH are not fully understood, but traditional risk factors are thought to explain only part of the increased risk (29, 30). Among HIV-infected individuals, the presence of obstructive CAD was associated with lower nadir CD4^+^ T-cell count and longer treatment with ART, similar to previous findings (31). They are both markers of longer duration of HIV infection with potential adverse metabolic effects. In addition, studies have shown that while antiretroviral drugs inhibited viral replication and led to increased longevity in HIV populations, they also increased cardiovascular risk (32, 33). In addition, the current CD4^+^ T-cell counts are used to monitor HIV infection status and the efficacy of ART (5), emerged as the third most predictive feature after age and total cholesterol. Furthermore, the CD4^+^ count < 500 cells/mm^3^ has been shown to be an independent risk factor for CAD, and its attributable risk is equivalent to traditional CAD risk factors (34). In some previous studies, the CD4^+^: CD8^+^ ratio, a marker of immune senescence, has been predictive of cardiovascular events, which is consistent with our study (35, 36).

The model containing only CACS (CACS model) had limited performance in predicting the prevalence of obstructive CAD in HIV populations. Compared with traditional models after adding the CACS, the Model 1 still showed the highest discriminative performance, which suggested that the Model 1 was able to reclassify CAD risk more accurately the PLWH population at low to moderate risk for obstructive CAD using only clinical information, avoiding the use of additional CACS. To further explore the incremental value of CACS, we constructed Model 3 by retaining the selected features and hyperparameters of Model 1 and adding CACS. The results showed that the model performance did not improve, with the AUC declining slightly and no statistically significant difference observed. These may be attributed to the relatively low burden of calcified plaques in asymptomatic PLWH (37), as well as potential collinearity between CACS and other variables such as age and lipid profile. These findings suggest that although CACS may be associated with CAD risk, it does not necessarily provide additional predictive value beyond a well-optimized model incorporating both traditional and HIV-specific clinical features. However, this finding still needs to be supported by prospective randomized trials with a larger sample size that could focus on assessing the importance of the coronary calcium score in the ML model.

CT contrast agents are more likely to produce drug allergic reactions in PLWH participants than the general population due to the continuous stimulation of immune cells in the HIV population (38). However, previous pretest probability models, including the CAD Consortium scores, GCM, and CRS, have had limited predictive power for HIV populations. The Model 1 with significantly higher AUC values performs well in terms of accuracy, calibration, and net-benefit (risk threshold from 0 to 80%). As a pre-test assessment (the clinical likelihood that HIV patients will have obstructive CAD), our model can avoid unnecessary coronary CTA in low (pretest probability <15%) and moderate risk (15% ≤ pretest probability≤70%) as much as possible (11). Given that all features used in our Model 1—such as age, lipid profiles, blood pressure, ART duration, and CD4^+^ counts—are routinely collected in HIV outpatient settings, this model is well-suited for clinical application. It may serve as a reference framework for developing clinical decision support systems (CDSS) to assist clinicians in pre-test risk stratification of obstructive CAD among asymptomatic PLWH (39). In addition, for patients with renal abnormalities or other contraindications to coronary CTA, the model can also be used as a reference for testing obstructive CAD to assist clinicians in evaluation and diagnosis and may help guide patient management. However, prospective studies with external validation and multi-center cohorts are needed to support its clinical integration and to evaluate its impact on clinical practice.

There are several limitations inherent to the present study. Firstly, due to the particular characteristics of the PLWH population, no external validation was conducted in an independent cohort in the current investigation. Although we have improved the algorithm, the actual clinical implementation and popularization of the model still need to be explored in further detail. Secondly, assessment of significant coronary stenosis was performed by coronary CTA without the use of invasive coronary angiography, although previous articles have demonstrated the high diagnostic value of coronary CTA for identifying coronary stenosis (40).

## Conclusion

The random forest model with the inclusion of HIV-specific metrics showed better performance and best fit compared with the traditional models. The machine learning model better predicted the likelihood of obstructive CAD in low to moderate risk PLWH and may be helpful for clinical decision-making.

## Data Availability

The raw data supporting the conclusions of this article will be made available by the authors, without undue reservation.
